# Showing the Unsayable: Participatory Visual Approaches and the Constitution of ‘Patient Experience’ in Healthcare Quality Improvement

**DOI:** 10.1007/s10728-017-0349-3

**Published:** 2017-10-16

**Authors:** Constantina Papoulias

**Affiliations:** Service User Research Enterprise, Health Service and Population Research, Institute of Psychiatry, Psychology & Neuroscience, King’s College London, De Crespigny Park, London, SE5 8AF UK

**Keywords:** Visual methods, Participatory methods, Patient experience, PhotoVoice, Experience Based Co-Design, Healthcare quality improvement

## Abstract

This article considers the strengths and potential contributions of participatory visual methods for healthcare quality improvement research. It argues that such approaches may enable us to expand our understanding of ‘patient experience’ and of its potential for generating new knowledge for health systems. In particular, they may open up dimensions of people’s engagement with services and treatments which exceed both the declarative nature of responses to questionnaires and the narrative sequencing of self reports gathered through qualitative interviewing. I will suggest that working with such methods may necessitate a more reflexive approach to the constitution of evidence in quality improvement work. To this end, the article will first consider the emerging rationale for the use of visual participatory methods in improvement before outlining the implications of two related approaches—photo-elicitation and PhotoVoice—for the constitution of ‘experience’. It will then move to a participatory model for healthcare improvement work, Experience Based Co-Design (EBCD). It will argue that EBCD exemplifies both the strengths and the limitations of adequating visual participatory approaches to quality improvement ends. The article will conclude with a critical reflection on a small photographic study, in which the author participated, and which sought to harness service user perspectives for the design of psychiatric facilities, as a way of considering the potential contribution of visual participatory methods for quality improvement.

## Introduction

Patients’ accounts of their treatment and overall engagement with services, or ‘patient experience’ data, are increasingly recognised as a key element in healthcare quality assessment, and an indicator of the strengths, weaknesses and overall usability of health care services and systems. Furthermore, there is considerable evidence that patient experience positively correlates with the other indicators of healthcare quality: patient safety and clinical effectiveness [15]. Therefore, closer attention to patient experience, as well as on-going collaborative work with patients, is seen as central to continuous improvement and to the development of responsive and sustainable healthcare environments [1]. The Care Quality Commission, the public regulator of health and social care services in England, stipulates that “providers must seek and act on feedback from people using the service, those acting on their behalf, staff and other stakeholders, so that they can continually evaluate the service and drive improvement” [20]. Moreover, the contributions of service users and carers to service reform are not to be limited to provision of feedback: the UK Health and Social Care Act 2012 stipulates that the National Health Service must ensure patient participation—both in individual care and in the commissioning of new services or other changes in existing provision [12].

However, there are considerable challenges both in the understanding of what constitutes ‘patient experience’ in services and in incorporating an effective engagement with such experience in quality improvement designs. So far, the concept of ‘patient experience’ is understood with reference to the literature of experiential marketing^1^ where it originates, and whence it has been transplanted to healthcare policy [42]. A recent narrative synthesis of conceptualisations of patient experience in health services research has shown that these can be summarised as attempts to enrich definitions of ‘consumer experience’ through references to the ethics of patient-centred care. The review thus defines ‘patient experience’ as the sum total of users’ episodes of contact with a service (‘touchpoints’), their attendant cognitive, emotional and sensorial responses to these episodes, the relationship of these responses to their expectations of the service [30] and, finally, how these episodes and their evaluation relate to the principles of patient-centred care [63]. This definition foregrounds the multimodality of the construct of patient experience, its processual nature and the heterogeneity of interactions with a service it is intended to embrace. In this context, the process of parsing out and measuring indicators for patient experience becomes particularly challenging [31].

Furthermore, a recent systematic review of approaches to patient experience data in quality improvement projects has identified persistent difficulties in integrating such data in service redesign [19]: data lack sufficient specificity; frequently, the aspects of a service targeted for improvement are those already highlighted by staff rather than those uniquely raised by patients; finally, improvement efforts which make use of patient experience typically target administrative practice (for example the booking of appointments) and do not tend to consider the relationship between clinicians, staff and patients, or broader organisational culture issues. These findings would suggest that a more consistent use of qualitative approaches to eliciting patient experience may address some of these difficulties, since such methods are better attuned to the processual and relational dimensions of patients’ engagement with services [45, 57].

In this context, this article considers the strengths and potential contributions of a particular orientation within qualitative practice, namely participatory visual methods, for healthcare quality improvement research. It argues that such approaches may enable us to expand our understanding of ‘patient experience’ and of its potential in generating new knowledge for health systems. In particular, they may open up those dimensions of people’s engagement with services and treatments which may exceed both the declarative nature of responses to questionnaires and the narrative sequencing of self-reports gathered through qualitative interviewing. Visual participatory methods may do so by bringing to the fore unattended dimensions of such experience—that is, the kinds of tacit knowledge or felt engagements that constitute our immersion in daily habits and routines. Additionally they may occasion an interruption of such immersion and an unsettling of the habitual ways in which we engage with patient experience data. The question I will engage here is not how we generate valid knowledge out of visual data or how we make patient experience usable in the process of quality improvement. Rather I will suggest that working with participatory visual methods may productively challenge our assumptions about the role of evidence and of patient experience in quality improvement work and—in so doing—might allow us to reframe how and for whom such experience or indeed ‘improvement’ is constituted.

To this end, the paper will first consider the emerging rationale for the use of visual participatory methods in improvement research more generally in order to outline the understanding of both ‘experience’ and the visual underpinning such rationale. As the term ‘visual participatory methods’ covers a broad range of approaches, this paper will focus on how photography and—to a lesser extent—video, have been used in health research more broadly, while also considering their potential role in quality improvement. In so doing it will examine how ‘experience’ emerges through visual means in two historically distinct, albeit related, enterprises, photo-elicitation and PhotoVoice, both of which are currently in use in health research. While the term PhotoVoice has catachrestically come to designate both research designs—with an attendant implication that they are both working within a similar participatory paradigm—I will discuss the two separately, while acknowledging that their boundaries are permeable. This is because, as I will argue, the two methods frame distinct research objectives and may also instantiate a distinct conceptualisation of experience, of the function of images in relation to that experience and of the role of participatory elements in the research process.

The paper will then move to a recently devised participatory model for healthcare improvement work, Experience Based Co-Design (EBCD). It will argue that EBCD exemplifies both the unique strengths and the potential difficulties of using visual participatory methods in quality improvement: it uses visual media (in this case video recordings) to ensure that a direct and intensive engagement with patient experience is located at the very core of improvement efforts, thereby potentially challenging normative organisational habits of ‘doing improvement’. However, by doing so, it may occlude how normative expectations about what ‘patient experience’ is may continue to structure the production and the consumption of narratives through these visual recordings. Finally, the paper will end on a critical reflection on a small study, in which the author worked as a member of the research team, and which made use of photography to harness service user perspectives on the design of healthcare facilities, as a way of opening up the challenges of bringing together the visual and the participatory for quality improvement.

## Participatory Photography in Quality Improvement: An Appropriation

Visual/photographic approaches have been steadily gaining ground in health services research in the last 20 years; even so, they represent a small part of qualitative research designs, while their use in quality improvement contexts is sparse. This is, in part, because of an unease surrounding the evidentiary force of visual methods for social science more broadly: images, photographs in particular, are characterised by an ‘ambiguous ontological status’; that is, they both reflect and refract that which they make visible, appearing too obvious and too ‘messy’ at the same time. They thus appear incommensurable to scientific inquiry [7, 33].^2^ This unease may be more marked in improvement research, where current priorities demand the abandonment of what may be seen as ‘intuitive’ or inductive approaches, in favour of working with standardised data, generating robust evidence-based models and producing generalisable and transferable knowledge [35, 54]. In this context, accounts of the usefulness of photographic methods for improvement research have to perform a delicate balancing act: they need to demonstrate the ability of image-based research to generate more in-depth data than other qualitative approaches, while also assuaging disciplinary suspicions concerning the validity and scientific value of such research. This balancing act is usually performed in three inter-related ways. Firstly photography-based approaches are positioned as supplementary and supportive to more robust means of evidence production. Thus, photographic data are put in the service of triangulation, where they are validated through their convergence with more conventionally generated quantitative and qualitative findings [3]. For example, two studies of in-patient perspectives on the design of healthcare facilities—including one in which the present author was part of the research team—used patient generated photographs alongside other methods (focus groups, interviews and postal surveys): their findings, reported via synoptic results’ sections and tables, were shown to be thematically consistent with those generated through the application of other methods (for example, participants valued the quality of design, accessibility, human–environment interaction and personal space). While findings unique to these methods were also reported, these were seen as supplementary to more conventionally generated results—their potential dissonance from such results underplayed [11, 14].

Secondly, the judicious application of photographic methods is seen as capable of addressing specific challenges that beset quality improvement research and, in so doing, of providing more auspicious conditions for its implementation. Thus, outcome reviews for participatory visual methods in quality improvement studies stress their potential in alleviating challenges around recruitment and retention (visual methods are more likely to engage seldom heard groups); their contributions to data quality (they may enable a better understanding of patient priorities); and their advantages for implementation and sustainability (their circulation may facilitate collaboration between different stakeholder groups) [3, 6, 25, 41].

Finally, concerns about photographs’ unruliness may be further assuaged through claims that they can act as a kind of ‘reality check’ for more established research methods [32]. There is an assumption here that the viewing of participant-produced photographs can lead to a ‘more direct understanding of people, their life experiences and their perceptions’ [48], enabling researchers and academics to empathetically ‘see the world through [the participants’] eyes’ [8].

Taken together, such arguments may facilitate the introduction of participatory photography in health improvement research. They do so at considerable cost, however: by rendering the visual compatible with more established methods, they elide the distinctiveness and representational complexity of photographs and the extent to which their meaning and impact is necessarily constituted by shifting pictorial conventions and social modes of seeing and by the precise conditions of production and reception in which they emerge [50]. Furthermore, the demand for validation and compatibility re-visions the primary motivation for engaging participatory methods, which is to elicit participants’ *own* ways of knowing. Such a demand implies that different ways of knowing can only become intelligible through the existing frameworks of research and of quality improvement. It thus apprehends attending to *different ways of knowing* as giving consideration to a *more diverse set of statements* about such improvement rather than as offering a potential reconceptualisation of knowledge production. Unlocking the potential of visual participatory methods by contrast means attending to photographs’ ‘ambiguous ontological status’, while embracing a participatory approach entails maintaining a consistently reflexive stance, that is to say, an alertness to the processes through which knowledge is produced and in particular to the relationship between knowledge and power. The demand for triangulation, by contrast, obscures the analysis of such relational dynamics, thus effectively silencing reflexivity [59].

## Experience as Felt Engagements: Photo Elicitation

Photo-elicitation is a technique developed in the 1950s with the aim of enhancing interview data in qualitative anthropology and sociology, initially with no participatory element. Its founding premise was that if researchers present participants with photographs related to their topic, then responses are more likely to be accurate and stay on topic [9, 21]. Furthermore, photographs could act as detailed records of a space or event, thus jogging participants’ memory so that they could produce more in-depth information. Participatory forms of photo-elicitation (‘autodriving’ or ‘reflexive photography’), are based on the additional claim that if participants take their own photographs, the ensuing interviews are more likely to be structured around their concerns, thus giving them the opportunity to ‘drive’ the project [24]. In view of its engaging and assistive potential, the use of participatory photography has provided a passport for the investigation of the living conditions of socially marginalised groups, particularly homeless people or those with a socially stigmatised health status [40]. In the context of public health research, photo-elicitation techniques have also been used to elicit the perspectives of those participants for whom the spoken word may not be the most effective means of communication: people living with dementia, young children, people with intellectual disabilities or those living with the effects of stroke [23, 27, 38, 53].

However, researchers using photo-elicitation have also maintained that working with photographs may enable participants to communicate aspects of their lifeworld which may not easily lend themselves to verbalisation, such as emotional expression [18] or tacit knowledge (know-how) [36] thus yielding a different range of data and potentially redefining the scope of empirical research [41]. For example, Baker and Wang used photo-elicitation techniques to investigate older adults’ experience of living with chronic pain: images of a knife, bottles of pain medication and a rose bush punctuated participants’ stories and provided a way for them to communicate the violence, continuous presence and invisibility of their pain [2]. A study on the function of hope for people with a diagnosis of schizophrenia found that participants who took photographs were more able to relate an abstraction such as hope to their everyday activities than those who did not. Thus, one participant used a photograph of a spoon to talk about how they found hope through their ability to engage in ‘banal’ daily routines such as using a spoon to eat an egg [37]. Finally, a photo-elicitation study of parents’ accounts of distress over their child’s preterm birth suggested that distress may not be experienced as an internal emotion, but as a concrete event located in specific objects (oxygen tanks, sanitising agents, medical records and even the body of their child). Such objects, including the child itself, may then be avoided by the parents in an attempt to control the unbearable aspects of that experience [28].

These examples amplify and complicate the conceptualisation of experience and, in addition, the status of the visual as evidence [22]. A photograph of a knife visualises something about the unshareable intensity of pain through a shared cultural metaphor; a photograph of a spoon stands for a ritual which gives the photographer a sense of stability; taking a photograph of the oxygen tank enacts a distancing from and a commemoration of the distressing experience of using the tank. Participants make photographs in an attempt to render an aspect of their experience communicable—the subjective intensity of pain, the performance of daily routines, the way in which objects elicit memories. However, that communication resides neither in what the pictures show nor in what the accompanying narratives say but in an elusive relationship between the two. Moreover, that communication effected through a process of isolating and framing particular objects or moments performs a kind of breaching or an interruption of ‘the taken for grantedness of [participants’] lives’, thus creating an outside from which to look at their lives anew [46].

## Experience as Collective Emergence: PhotoVoice

If photo-elicitation represents an enhancement of the depth of qualitative data and an opportunity to include tacit knowledge and felt engagements with the world as data, PhotoVoice proposes instead an iterative and collective articulation of experience and a challenge to the power relations which characterise the research process. Unlike photo-elicitation techniques, where photographs are used in the context of a conventional one-on-one encounter between participant and researcher, PhotoVoice is a methodological innovation within Community Based Participatory Research (CBPR) and, as such, uses photo-elicitation techniques in the context of extensive collaborative work in the pursuit of social justice.

Most forms of CBPR draw their principles from the work of Brazilian educator Paulo Freire, who developed a roadmap for action education for marginalised indigenous communities. Freire’s work aimed to create the possibility for what he called ‘praxis’, that is, informed action directed at challenging the socio-political conditions of marginalisation [17]. CBPR is not a research method as such, but an ethical orientation which seeks to shift control of the research agenda setting, process and goals from the researchers to the ‘researched’, thus reducing the power inequalities which structure work in conventional social science settings, while simultaneously shifting the goals of research from the generation of knowledge to the facilitation of change according to local priorities and needs [60]. This shift of control is enabled, in part, through a legitimation of local knowledges and native conceptual frameworks. In this context the spoken word is not the only or the main channel of communication: rather, local symbolic resources are preferred where possible, since they encapsulate the means by which such knowledge is produced and made intelligible to the community in question. These resources often amount to visualisations such as body maps, calendars or performance [10].

PhotoVoice refocuses the practice of CBPR towards translatability—photography becomes a means of translating local concerns into a community ‘voice’ which in turn becomes legible to a wider audience of policy makers and clinicians. This method was initially developed by anthropologists Wang and Morris to aid the assessment of the reproductive health of women in rural China in the early 1990s [64]. Wang and Morris did not make use of local symbolic resources, and instead trained their participants to take photographs with which to record their living conditions. In their initial formulation, PhotoVoice approaches (with ‘-*Voice*’ functioning as an acronym of ‘*V*oicing *o*ur *i*ndividual and *c*ollective *e*xperience) had three aims: to enable local communities to represent their own concerns; to promote reflection on these; to empower these communities to enter the policy conversation [62]. Wang and Morris argued that photography was well equipped to promote these aims because it provided an accessible and readily useable symbolic resource to people of non-dominant literacies; furthermore it could act as a switch point, allowing local concerns to become visible to decision makers. However, photography does not simply function as a means of illustrating living conditions (e.g. by showing the distance travelled by village women to access drinkable water). Rather, it became an instrument facilitating deliberation. Photography assisted the iterative emergence of a community ‘voice’, through a very precise staged procedure articulated in Wang and Morris’ initial presentation: after participants take a number of photographs, community members and researchers come together to *select* which photographs to use for their needs; they then specify and *contextualise* their selections by discussing the significance of the photographs and the purpose they will serve; lastly participants and researchers together *codify* these discussions, by reflecting on participants’ narratives and coming up with themes and patterns to express through them [61]. Thus we could say that PhotoVoice aims to create the conditions for social change by generating the space for participants to critically reflect on, and thereby collectively reframe and politicise, their immediate shared concerns and challenges (for example a lack of access to clean water) so that they may come to see them as instantiations of their social and economic marginalisation. Rather than suggesting that photography can enable a direct expression of community needs, this reflexive and deliberative dimension of PhotoVoice proposes that collective engagement with photographs can provide the means for the emergence of something that was not there before. In this sense, ‘experience’ and ‘voice’ do not refer to a set of community practices which are simply made visible in photographs, but rather to a process of coming-to-collective-awareness generated through analysing such practices anew. Such coming to awareness might be construed as a process of ‘making the familiar strange’ [34]—as a disruption of habitual narratives in which communities find their collective identity.

Insofar as the process of PhotoVoice enables a re-interpretation of the community health needs and priorities, we could then see such a process as a recasting of photo-elicitation within a horizon of social justice. However, unlike conventional photo-elicitation methods, PhotoVoice projects purport to empower participants by constituting them as collaborators rather than informants or research subjects. In so doing, they also raise questions about the ethics and reach of participatory methods. These are questions about the extent to which interpretations of photographs produced under conditions of inequality can be construed as ‘shared’ or ‘mutual’ and come to function as evidence and a vehicle for a ‘voice’ for the community [43]. These conditions of inequality refer to the stratification of local voices through gender, age and other markers of status which will determine who is licensed to speak, about what and under what conditions. They also refer to the fact that PhotoVoice projects are typically initiated by metropolitan researchers and conditioned by the resources, design, timescale and overall aims of research institutions and their funders. Furthermore, the structural inequalities between researchers and participants inform not only how that voice is constituted but also the contexts in which it may circulate [39]. Indeed, a 2016 meta-analysis of PhotoVoice projects found that involvement of local participants in the interpretation and dissemination of PhotoVoice findings is somewhat limited, while a 2014 systematic review found that, while PhotoVoice presents a powerful tool for addressing social inequalities and marginalisation, its potential is rarely actualised in the form of interventions leading to social change [16, 52].

These questions, however, are not unique to PhotoVoice projects. Rather they challenge the claims around empowerment which define participatory research more broadly. In so doing they point to an ethical and epistemological requirement for ongoing reflexivity within the research process: that is, an attentiveness to the conditions through which knowledge and voice are generated and to the kinds of exclusions that may structure both. This would mean an attentiveness to the discursive and contextual processes through which photographs are made to speak [65] and to the extent in which such ‘speech’ may work to unsettle not only community narratives but also institutional knowledges and priorities.

## Patient Experience as Visual Testimony: Experience Based Co-Design

Having discussed the potential richness of participatory photography for quality improvement, I now turn to a framework which is currently gaining purchase in the collaborative design of healthcare environments. Experience Based Co-Design, (hereafter EBCD) uses film rather than photography; nevertheless, I am considering it here because it is a visual participatory framework that has been specifically developed for healthcare quality improvement work and, as such, aims to maximise the usability of patient experience for improvement. While filmed patient narratives constitute only one part of EBCD, I would argue that visual presentation plays an important role in this maximisation. Therefore, I will consider here some of the epistemological and ethical consequences of this methodological choice.

EBCD is essentially a hybrid design, however its main filiation is from design theory whence it borrows both the definition of user experience and that of collaborative practice. Developed in the UK by Paul Bate and Glenn Robert, EBCD works on the premise that good design is user centred and mindful of “how well people understand [the service], how they feel about it while they are using it, how well it serves its purpose, and how well it fits into the context in which they are using it” [4]. Most EBCD toolkits describe six interlocking phases (although numbers may vary) all of which (at least nominally) operate through a collaborative dynamic [56]: (1) introduction; (2) gathering staff experiences of the service in question; (3) gathering patient and carer experiences of the service; (4) bringing staff, patients and carers together to identify shared priorities for improvement; (5) co-design workshops; (6) end of process and celebration. The gathering of staff experiences may involve a variety of methods, such as interviews and observations, while work with patients and carers typically centers around filmed interviews. These interviews are thematically analysed by the research team who identify ‘touchpoints’, that is, recurrent, emotionally salient moments which will constitute potential areas for improvement. The research team then produce a short edited video in which the identified touchpoints may be used as intertitles structuring together fragments of different participants’ narratives. Patient and carer participants view the film together, in order to further clarify and refine the touchpoints, thus honing their priorities for the next stage in the process. This is followed by a joint meeting between staff, patients and carers which makes use of the film to ‘trigger’ discussions and set the agenda for the ensuing collaborative re-design practice [49]. Initially tested in a UK head and neck cancer service in 2005, EBCD was in use in 59 projects worldwide by the end of 2013, with work reportedly becoming potentially more geared towards patient priorities [13]. In a multisite evaluation of a series of EBCD interventions in Emergency Departments across Australia, Piper and colleagues found that the use of this framework tends to promote better understanding between clinicians, carers and patients and that shared viewing of patient narratives may bring ‘the dynamics of care’—that is, the relational aspects of service delivery—more into focus for improvement research [44]. Indeed, we could thus argue that EBCD is a flexible and capacious approach capable of reworking the principles of visual participatory methods and of PhotoVoice in particular, to fit the requirements of improvement projects. Like PhotoVoice, EBCD makes use of visual materials not as ends in themselves, but as a means by which to create a space for reflection and, through it, a collective voice—in the form of shared priorities for improvement. Furthermore, in making use of the film to instigate a co-design process, EBCD arguably takes the participatory principles further than some PhotoVoice projects by moving beyond priority setting to engage patients as *actors* in a collaborative shaping of service improvement interventions. Certainly, the potential success of EBCD work hinges on the receptivity of the institutional context in which it plays out: the potential for such work to undercut institutional priorities, professional hierarchies and their attendant exclusionary force depends on ‘co-design readiness’; and institutional ability to tolerate the possibility of ‘dialogical innovation’ [26].

Admittedly, the filming and shared viewing of patient and carer narratives is only one aspect of the overall design of EBCD interventions; indeed the later phases of the framework move away from the film to concentrate on the process of co-design. Nevertheless, I would argue, the identification of emotional touchpoints and the viewing of the film are key moments and act as launchpads for the co-design process: accounts of EBCD typically stress the importance of placing patient experience at the centre of improvement efforts and both the production and the shared viewing of the film are the means by which such experience is rendered intelligible for the purposes of collaborative work. Indeed, published accounts of EBCD projects converge on a claim that film can enliven patients’ accounts, convey a sense of emotional authenticity and cultivate clinicians’ empathy [29, 58]. This linking of filmed narratives to authenticity is problematic however. The film’s syntax is—for the most part—the researchers’ own work: while patients play a critical role in the identification of areas for improvement, they are typically absent from the editing process. Admittedly this absence is not necessitated by the structure of EBCD; indeed some projects have taken a more collaborative approach to editing [55]. Perhaps more fundamentally however, the use of film to generate empathy by amplifying patients’ narratives may paradoxically limit the potential of visual methods to provide new means of understanding patient experience. This is because EBCD, by focusing on patients as talking heads, relies on the physical presence of the speakers to guarantee the integrity or moral value of their statements—we take their statements at face value because they function as testimonies. Thus the trigger video may work to hypostatise patient narratives: by focusing on the person who discusses their experience of a service or treatment, it becomes a way of asserting the ‘truth’ of what patients are saying, rather than serving as an instrument through which new ways of seeing may be elicited.

An alternative way of using film in quality improvement projects may provide a useful counterpoint here. A study of work practices in an Australian intensive care unit made use of what researchers called ‘video-reflexive ethnography’ with the aim of assessing and re-designing clinical communications [5]. Here, video interviews with staff were supplemented by extensive filmed observations of structured activity in the unit. The research team then edited footage of ward rounds, planning meetings and an interview and presented the resulting film in a discussion session (also filmed) with the clinicians and staff. The authors argued that this process enabled their participants to grasp the complexity and multi-layered nature of communication in the ward in ways that had not hitherto been available to them. By training the camera towards the interactions which are constitutive of the participants’ working routines, the project used the visual to unsettle and interrupt participants’ immersion into their working lives. It thus produced a new ‘structure of attention’ bringing to the fore aspects of such routines which normally remained below the threshold of perception.

## The Constitution of Patient Experience in a Photographic Study of the Design of Psychiatric Wards: A Reflection

For the remainder of this paper, I will turn to a brief photo-elicitation study of service user perceptions of healthcare facilities in which I was part of the researcher team. The results of this study have been reported elsewhere [2]—this discussion is not meant as a demonstration of how to work with visual participatory designs for quality improvement projects. Our findings are sparse and can only be used indicatively: time and budgeting constraints as well as ethical and clinical considerations prevented us from staging more extended or systematic encounters with participants. Rather, this is a reflection on how participatory photography might work if untethered from the requirement to provide the kinds of evidence that would support and supplement more conventional methods. Such work, I would suggest, may trouble our understanding of how empirical evidence is constituted, since our findings may not be contained in the photographs nor in the accompanying words, but may be brought into being in a shuttling between words and images. Additionally, I wish to consider how a more reflexive reading, one which focuses on the encounter between researcher and participant, may reorient and enrich our understanding of how participants may be using photographs in this project and in quality improvement research more generally.

*Design in Mind* was a mixed methods study which aimed to elicit mental health service user perspectives of the design and physical environment of acute psychiatric wards in an inner city environment. The study used a participatory model, in which service user researchers work together with groups of local service users to generate measures for the evaluation of healthcare environments or treatments [51]. Additionally, and since the emphasis in this project was on the physical aspects of the wards, photographs taken by in-patients were employed as supplementary data. In accordance with emerging evidence of the potential usefulness of photo-elicitation for health services research, we hypothesised that photography would engage and stimulate participants and allow us to negotiate language barriers. Furthermore, we wished to make use of photographs to triangulate in-patient priorities generated through the qualitative work (interviews) which had led to measure generation. People recruited for a feasibility study of the measure in two of the inner city acute wards involved were also invited to take two photographs: one of something they liked and one of something they did not like about the ward. Since the sites were locked psychiatric wards, where the majority of in-patients were detained for compulsory treatment under the Mental Health Act, regulatory restrictions concerning health and safety and confidentiality shaped the photo-elicitation procedure. Thus, cameras could not be left with participants, photographs could not include people and the time spent with each participant was limited. Participants who consented were given a point and click camera and the researcher accompanied them across the ward and recorded the reasons they gave for their choices. Their responses were then transcribed verbatim and thematically analysed, while the frequency with which different parts of the ward elicited positive or negative responses was also examined.

In the event, 70 photographs were produced. Most of the positive photographs were taken in bedrooms and common rooms while, overwhelmingly, negative photographs were taken in the shared bathrooms. Emerging themes included a need for control, a valuing of both privacy and sociality, the association of shared facilities with lack of hygiene, a frustration around inaccessible or barred spaces. In accordance with our predictions, these themes were also shared across interview data and free text boxes in the piloted measure. However, some (albeit minor) findings specific to the photographs also emerged: photos of light switches outside the bedroom door, of a shower button and of a one-way door lock were discussed in terms of disruption to daily routines (inability to read in bed, interrupted water flow, being locked out of the bedroom when using the toilets). Photos of integrated toilet and shower facilities, of a lumpy bed and of windows covered in privacy film, were accompanied by participants’ references to rooms and objects which were not what they were supposed to be (the showers did not clean, a bed did not allow for rest, windows had no view). In these cases, participants used photos to demonstrate how the spatial organisation and fixtures and fittings of the wards disrupted their daily routines and blocked the flow of activities in the ward. Their reasons for disliking these spatial arrangements thus appeared to relate to bodily impingements and to the manner in which such arrangements upset participants’ expectations about the zoning and separation of functions which constitute a livable space (washing should be separate from evacuating; windows should be offering views). Additionally, constraints specific to the study (the fact that the researcher had to follow participants as they took photos), potentiated participants’ responses: while taking photos, participants moved around or invited the researcher to do so in order to make their discomfort communicable. On these occasions, it was the engagement with the researcher as a physical presence in the ward rather than the content of the images that may have articulated the ‘patient experience’ of the ward space.

Additionally, some of the positive photographs were accompanied by accounts of imagined or remembered spaces: a photograph of the living room was discussed in terms of forgetting the ward through watching TV; a view of the grounds from a bedroom window occasioned a reverie of demolished buildings and gardens which had stood there when the participant had first been admitted 20 years previously; a woman took a photo of her fitted wardrobe and spoke fondly of hotel rooms and the holidays of her youth. Here, what was liked was not something shown in the photographs but, arguably, something imagined in the act of taking a photograph, and possibly conditioned by the presence of the researcher, an act which also afforded a disengagement and a means of escape from the here and now of the ward.

The above examples may all be contained under our existing theme of ‘control’. However they may also provide an alternative way for us to conceptualise patient experience. The process of taking photographs may enable a distancing of the participant from the space thus framed—thereby precipitating a momentary breach which ‘unhing[es] the viewer from their thus-far taken-as-given life world’ [5]. Such a breach may enable participants to articulate felt engagements with—in this instance—the physical space of healthcare facilities. In this sense, priorities for improvement may not only be identified through *what* the participants are showing (dirty toilets and gardens, empty or locked activity rooms) but may also depend on *whom* they are showing it to and how. Furthermore, photos of window views, TV rooms or fitted wardrobes may not demonstrate which fixtures and fittings are valued by the patients but, rather, how people use imagined or absent spaces (a holiday resort, a demolished building, or the flow of images on TV) to mediate and alleviate the distress associated with being an in-patient, particularly in the context of compulsory confinement [47].

A more reflexive reading might allow us to interrogate such findings further. In this project, where equal numbers of men and women participated, men were three times more likely to take photographs of their bedrooms. A conventional response to this may consider whether men value privacy or withdrawal. However, we may also attend to the gender dynamics of the encounter between researcher and participant: after all, taking these photographs presented an opportunity for male participants to take the researcher (whom they read as female) to their bedroom and to keep them there for a short time. We might consider how this act might constitute a response to the regulatory environment of psychiatric wards in which different conditions of accessibility apply to different users: normally visitors, including researchers, are allowed access to some (or all) of public areas only. In this case, the requirement that the researcher accompany the participants may have presented an opportunity for the latter to overwrite such spatial regulations while also introducing a dimension of solicitation to the encounter. A comparable dynamic may have motivated participants’ overwhelming preference for photographing bathrooms and toilets. By persistently pointing the camera at certain distressing objects—an overflowing toilet for instance—participants not only produced visual records to underscore the claims about unhygienic conditions in the ward, they also ensured that the researcher (who, unlike most participants, was free to come and go as they pleased) would stay locked in the toilet with them, their mobility and freedom as curtailed as theirs had been. In these cases, the process of making the photographs may have enabled a momentary redistribution of power or even a brief role reversal between participants and researcher, and a breach in the regulatory space of the ward.

This tentative account may allow us to open up the category of ‘patient experience’ through reference to negotiations of the regulated spaces of hospitals and healthcare environments, as redistributions of power enacted between staff and service users; finally such an account might allow us to consider how the here and now of healthcare environments may be shot through with remembered or imagined spaces.

## Participatory Visual Approaches and Quality Improvement: An Uneasy Fit?

This paper has used examples of participatory photography and video to suggest that participatory visual methods have the potential to broaden the scope and relevance of patient experience data for healthcare quality improvement. By enabling participants to produce data themselves, such methods may introduce topics and perspectives not anticipated by researchers. Photo-elicitation techniques may enable us to access symbolic and sensory aspects of experience which may not emerge in written reports or interviews with patients. Training a camera on the spatial articulation of healthcare facilities or on objects and interactions which constitute particular treatments or interventions can emphasise what had hitherto remained un-noticed and make visible some of the affective and social complexity of our engagements with health services. Equally, PhotoVoice work might make use of participant photographs to facilitate deliberation and generate new and collective articulations of patient centred healthcare. Thus, in the context of healthcare quality improvement, PhotoVoice techniques may force us to challenge institutional priorities about what constitutes feasible or desirable service change. The growth and success of Experience Based Co-Design projects, which use comparable techniques to engender a shared language of priorities for improvement, testify to the potential of visual participatory work in this field.

However, as we have already suggested, engaging with participatory photography and film is likely to demand unsettling established practices of evidence generation that have come to define quality improvement. Such engagement therefore requires an understanding that photographs and film afford a different way of knowing, the benefits of which are vacated when images are used to simply bolster or emphasise textual or numerical evidence. Furthermore, using participatory methods requires being open to new ways of working, and to re-examining institutional priorities and professional expertise. In this context then, I would suggest that taking participatory photography or film seriously within quality improvement will require specific attention to the methods, processes, and interpretative frameworks used both to analyse and to draw implications from these kinds of data. In the course of this paper, I have argued for the importance of acknowledging the social dimension of images: that making and presenting images is not simply about providing data about particular experiences, but simultaneously about enacting a relationship between participants and researchers, and about engaging with what is not there (temporally, spatially) as much as with what is denoted in the image. For this kind of approach to be robust enough to sit alongside other—more dominant, orthodox—ways of generating data for quality improvement, careful attention to a number of facets of research is required. These include how researchers are trained to employ and analyse participatory visual work; how research is organised [including power differentials with service users/patients]; and how very different kinds of data are brought together in multi-disciplinary contexts of evidence generation. The question then becomes what kinds of expertise do we need, so that we may attend to such engagements within quality improvement work and, moreover, how might we work collectively to facilitate methods and ethics that are able to receive—and do justice to—what can, all too often, remain unsayable?

## Footnotes

1 The marketing of products which focuses on how they might facilitate consumers’ relationship with a brand rather than fulfill their needs or address their desires.

2 This suspicion is all the more paradoxical given the constitutive role of visualisation techniques in the production of scientific knowledge and in health and medicine. However, a discussion of the history of medical or health related image production and the use of such images as evidence, is beyond the scope of this article.
